# Triglycerides/high-density lipoprotein-cholesterol ratio outperforms traditional lipid indicators in predicting metabolic dysfunction-associated steatotic liver disease among U.S. adults

**DOI:** 10.3389/fendo.2025.1591241

**Published:** 2025-04-15

**Authors:** Jin Yuan, Xuequan He, Yan Lu, Xuehua Pu, Lihe Liu, Xuejun Zhang, Jinping Liao, Guiling Li, Ying Luo, Tianwu Zhang

**Affiliations:** Puer Hospital of Traditional Chinese Medicine, Puer, China

**Keywords:** triglycerides, HDL cholesterol, National Health and Nutrition Examination Survey, cross-sectional study, ROC curve

## Abstract

**Objective:**

Triglycerides (TG), high-density lipoprotein cholesterol (HDL-c), and their ratio (TG/HDL-c) are key lipid markers associated with metabolic dysfunction. This study aims to investigate the association of TG, HDL-c, and TG/HDL-c ratio with metabolic dysfunction-associated steatotic liver disease (MASLD) and to assess whether TG/HDL-c ratio provides superior predictive ability for MASLD compared to TG or HDL-c alone.

**Importance:**

Although previous research has explored the relationship between TG/HDL-c and MASLD, the applicability of these findings across different ethnicities and populations remains uncertain. Additionally, this study is based on NHANES data, which relies on self-reported measures and lacks longitudinal follow-up, limiting the ability to establish causal relationships. While we adjusted for multiple covariates, residual confounding cannot be ruled out. Therefore, further large-scale, prospective studies are needed to validate these associations and assess the long-term predictive value of TG/HDL-c ratio for MASLD.

**Methods:**

A cross-sectional study utilizing the NHANES 2017-2020 database was conducted. We performed univariate and multivariate logistic regression analyses to examine the associations between TG, HDL-c, and the TG/HDL-c ratio with MASLD. Receiver Operating Characteristic (ROC) curve analysis was used to evaluate the predictive effectiveness. Sensitivity analysis was carried out using multiple imputation for missing data and subgroup stratification to validate the findings.

**Results:**

TG, HDL-c, and TG/HDL-c ratio were significantly associated with MASLD (p < 0.05 for all). The TG/HDL-c ratio demonstrated the highest predictive value (AUC = 0.732, 95% CI: 0.683–0.781), compared to TG (AUC = 0.713, 95% CI: 0.664–0.762) and HDL-c (AUC = 0.313, 95% CI: 0.264–0.362). The weak predictive power of HDL-c alone may be attributed to its complex role in lipid metabolism and potential confounding by other metabolic factors.

**Conclusion:**

Maintaining favorable levels of TG, HDL-c and TG/HDL-c ratio may lower MASLD risk. Using TG/HDL-c ratio could improve prediction models compared to individual TG or HDL-c markers.

## 1 Introduction

Metabolic dysfunction-associated steatotic liver disease (MASLD), a concept introduced in 2020, refers to a fatty liver disorder linked to systemic metabolic dysregulation (1). This novel definition and criteria aim to identify patients at increased risk for adverse health outcomes (2). Diagnosis of MASLD is primarily based on the presence of hepatic fat accumulation, which can be confirmed through liver biopsy, imaging, or blood biomarkers. Additionally, the condition requires the presence of one or more metabolic risk factors, such as overweight/obesity, type 2 diabetes, or other metabolic dysfunctions (3). MASLD has become a global health concern due to its rising prevalence, with significant regional variations in incidence rates. Epidemiological studies indicate that its prevalence is increasing over time, especially in high-risk populations such as those with obesity, type 2 diabetes, or metabolic syndrome (4, 5).

MASLD differs from the traditional concept of Non-Alcoholic Fatty Liver Disease (NAFLD) in that it more accurately reflects the pathophysiology of the disease, allowing for a more comprehensive and standardized approach to patient management (6–8). Unlike NAFLD, which primarily excludes other liver diseases, MASLD is defined based on metabolic risk factors, necessitating a reevaluation of predictive markers to improve risk assessment (9). As a multisystemic disease, MASLD can manifest in various forms, ranging from simple steatosis to liver fibrosis and even hepatocellular carcinoma. It also has extrahepatic manifestations affecting the cardiovascular system, non-hepatic malignancies, cognitive functions, and the respiratory system. The introduction of the MASLD concept thus links the diagnosis of metabolic dysfunction with risk factors for disease progression, excluding the influence of alcohol or other primary liver diseases, to more accurately assess the severity of fatty liver (4, 10, 11). MASLD significantly contributes to the healthcare burden, both in terms of direct costs, such as healthcare services and hospital admissions, and indirect costs like lost productivity and premature mortality. The global rise in MASLD prevalence, driven by increasing rates of obesity and type 2 diabetes, places a substantial strain on healthcare systems. In the U.S., MASLD has been linked to an elevated risk of liver cirrhosis, liver failure, and hepatocellular carcinoma, which add to morbidity and mortality rates. Furthermore, MASLD has been recognized as a major risk factor for cardiovascular diseases, further amplifying its public health impact. Current treatment strategies for MASLD focus on managing underlying metabolic dysfunctions, including insulin resistance, obesity, and dyslipidemia. While no specific pharmacological treatments have been approved yet, lifestyle interventions such as weight loss, dietary changes, and increased physical activity remain central to disease management. Early detection of MASLD is crucial, as the disease is often asymptomatic in its early stages, and timely intervention can prevent progression to more severe liver complications and cardiovascular events.

In recent years, the ratio of triglycerides to high-density lipoprotein cholesterol (TG/HDL-c) has emerged as a focal point of research in the academic field. Numerous studies have confirmed its independent association with cardiovascular diseases and metabolic syndrome. Unlike the solitary measurements of TG or HDL-C, the TG/HDL-c ratio, as a comprehensive indicator of dynamic changes in lipid metabolism, not only reflects abnormalities in fatty acid supply and lipoprotein metabolic function but also indicates impairment in the reverse cholesterol transport pathway. Given that MASLD is closely linked to metabolic dysfunction, the TG/HDL-c ratio serves as a valuable marker by simultaneously capturing disturbances in lipid metabolism, insulin resistance, and hepatic fat accumulation—key mechanisms in MASLD pathogenesis. This ratio has been shown to be predictive of hypertension (12, 13), cardiovascular disease (14, 15), diabetes (16–18), metabolic syndrome (14, 19), hyperuricemia (20, 21), and chronic kidney disease (22, 23). Moreover, its close association with NAFLD has been validated in multiple studies (24–26). While some research suggests that findings related to NAFLD could be applicable to MASLD, direct evidence remains insufficient. Although studies have indicated an association between TG/HDL-c ratio and MASLD, supporting its role as an independent risk factor and potential diagnostic marker (27, 28), the current body of research is scarce. There is a critical need for further studies across diverse populations to establish the predictive value of TG/HDL-c ratio in MASLD with greater certainty.

This study hypothesizes that the TG/HDL-c ratio, as a composite lipid marker, has greater predictive power for MASLD than either TG or HDL-c alone. The novel aspect of this research lies in its use of a large, representative sample from the NHANES database to examine this relationship across various demographic groups. By comparing the predictive performance of TG, HDL-c, and the TG/HDL-c ratio, this study adds new insights into the clinical utility of lipid markers in identifying MASLD risk. It provides a foundation for future research on integrating composite lipid markers into routine clinical screening for MASLD.

## 2 Method

### 2.1 Study design and sample population

This study’s data were sourced from the National Health and Nutrition Examination Survey (NHANES) conducted by the Centers for Disease Control and Prevention (CDC) in the United States (U.S.) (29). NHANES employs a complex stratified random sampling strategy to ensure the representativeness of the U.S. population across various age groups, regions, and racial compositions. The survey includes health and nutrition-related household interviews, physical examinations, and laboratory tests. Notably, the 2017-2020 NHANES cycles included additional liver disease screening programs, assessing fatty liver through self-reported histories and biochemical marker tests. Given the high prevalence of MASLD and its strong association with other metabolic disorders and cardiovascular diseases, NHANES provides a robust and comprehensive dataset for studying MASLD in the U.S. adult population. The inclusion of detailed biochemical markers and lifestyle factors makes NHANES an appropriate and reliable dataset for evaluating the predictive efficacy of various indicators for MASLD in a representative population.

The study states that 13,095 participants were included, representing an estimated 241 million U.S. adults. To generate nationally representative estimates, we applied the NHANES survey weights according to the analytical guidelines provided by the National Center for Health Statistics. These weights account for the complex survey design, including oversampling of certain populations, non-response adjustments, and post-stratification to match U.S. Census population totals. Specifically, we utilized the mobile examination center (MEC) weights appropriate for the survey cycles included in our analysis. For pooled analyses across multiple survey cycles, we adjusted the sampling weights according to NHANES analytical guidelines by dividing the original weights by the number of cycles combined.

### 2.2 Definition of diagnostic criteria and groups

**Figure 1:**
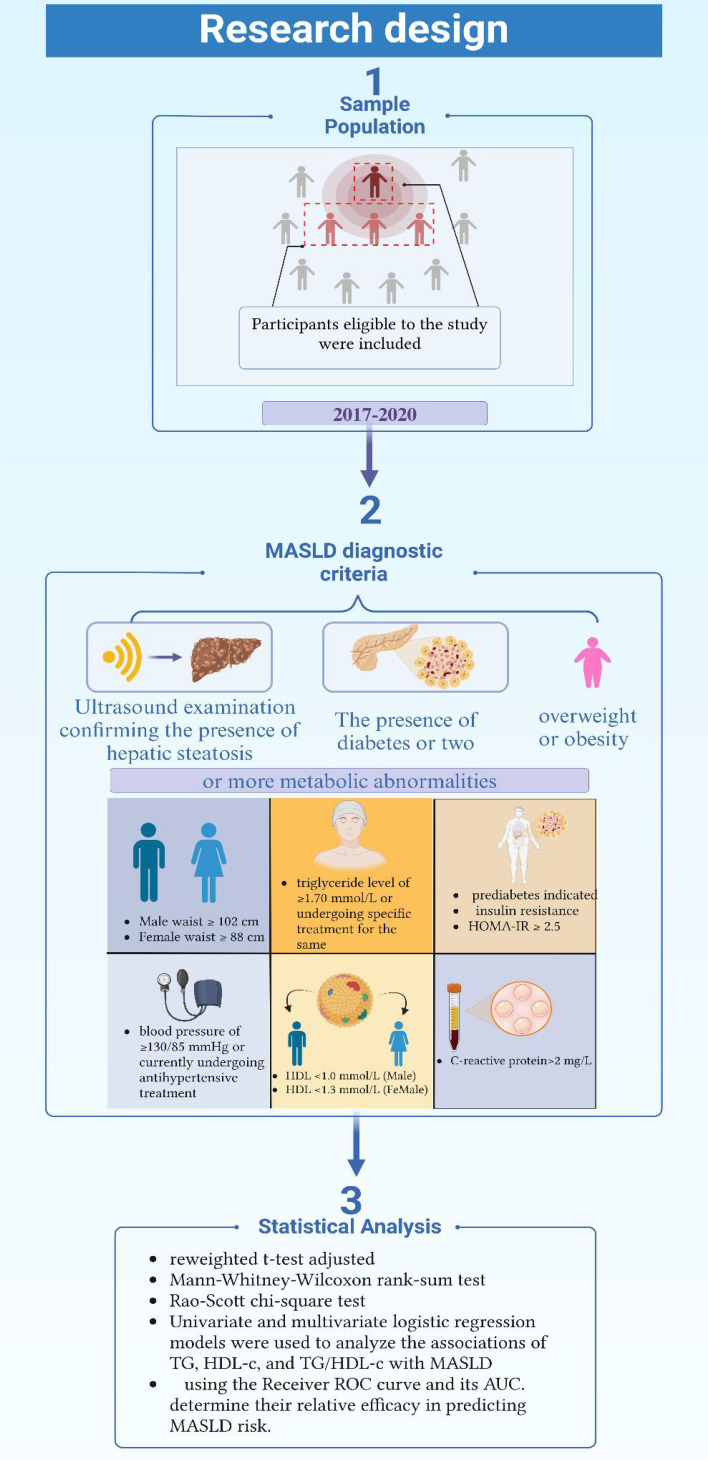
Research design and MASLD diagnostic criteria.

In this study, the diagnosis of MASLD required the simultaneous fulfillment of the following three criteria: 1) Ultrasound examination confirming the presence of hepatic steatosis; 2) The presence of overweight or obesity; 3) The presence of diabetes or two or more metabolic abnormalities. These metabolic abnormalities include: a waist circumference of ≥102 cm in men and ≥88 cm in women; a blood pressure of ≥130/85 mmHg or currently undergoing antihypertensive treatment; a triglyceride level of ≥1.70 mmol/L or undergoing specific treatment for the same; a high-density lipoprotein cholesterol level of <1.0 mmol/L in men and <1.3 mmol/L in women; prediabetes indicated by abnormal blood sugar levels; significant insulin resistance, indicated by a Homeostatic Model Assessment of Insulin Resistance (HOMA-IR) score of ≥2.5; and a C-reactive protein level of >2 mg/L. Participants who did not meet all the above criteria were classified into the non-metabolic associated fatty liver disease group (**Figure 1**) (30, 31).

### 2.3 Laboratory measurements

In the NHANES program, the processing of blood samples adheres to standardized procedures, which include immediate placement of the samples on ice after collection, followed by the separation of serum/plasma and freezing at -20°C, and then transportation to the National Center for Environmental Health (NCEH) laboratory in the U.S. All laboratory measurements were performed using the Roche Cobas 6000 chemiluminescent immunoassay platform with manufacturer’s quality control system and assay kits, following American laboratory accreditation standards.

Serum TG was measured using an enzymatic method based on lipase hydrolysis and subsequent glycerol phosphate oxidation reactions. HDL-c was determined using a direct immunoinhibition method, where antibodies against beta-lipoproteins selectively allow HDL-c measurement while blocking other lipoproteins. Detailed laboratory procedures and quality control protocols are available on the NHANES website.

In this study, standard protocols were followed to define and categorize the demographic characteristics of the subjects. Racial/ethnic categories were determined through self-completed questionnaires, and educational levels were classified into three categories: college or above, high school, and below high school or incomplete high school education. Body Mass Index (BMI) was calculated using the international standard for obesity, which involves dividing weight (in kilograms) by the square of height (in meters).

Alcohol consumption was categorized according to the traditional classification of the National Health and Nutrition Examination Survey in the U.S. Individuals were classified based on their self-reported average daily intake into categories: long-term non-drinkers (less than 12 times per year for both men and women), low-risk drinkers (less than 2 drinks per day for men and 1 drink per day for women), moderate-risk drinkers (2-4 drinks per day for men and 1-2 drinks per day for women), and high-risk drinkers (more than 4 drinks per day for men and more than 2 drinks per day for women).

Hypertension was determined using the adult blood pressure classification standard of a systolic pressure of ≥140mmHg or a diastolic pressure of ≥90mmHg, or if the subject was currently taking more than two types of antihypertensive medications. Diabetes history was ascertained through blood sugar indicators, glycated hemoglobin results, and whether the subject was currently taking more than two types of hypoglycemic drugs, dividing the study subjects into non-diabetic and diabetic groups.

Smoking status was categorized into three groups: non-smokers (less than 100 cigarettes in lifetime), successful quitters (cessation for ≥1year), and current smokers.

### 2.4 Statistical analysis

The statistical analysis in this study thoroughly considered for the weighting, clustering, and stratification features of NHANES’s complex sample design. All subjects included in the final analysis were ensured to have sufficient information for determining the presence or absence of MASLD. Depending on the normal distribution characteristics of continuous variables, different testing methods were adopted: for normally distributed weighted continuous variables, an improved variance estimation formula and a reweighted t-test adjusted for the complex sampling design were used; for weighted continuous variables not following a normal distribution, the Mann-Whitney-Wilcoxon rank-sum test was employed; for weighted categorical variables, the Rao-Scott chi-square test was applied. This analytical approach adhered to NHANES’s standard practices for statistical analysis and adjustment methods for complex sampling designs.

Univariate and multivariate logistic regression models were used to analyze the associations of TG, HDL-c, and TG/HDL-c ratio with MASLD, with results expressed as Odds Ratios (OR) and their 95% Confidence Intervals (CI). The overall fit of the logistic models was evaluated using methods such as the Hosmer-Lemeshow goodness-of-fit test. The multivariate models adjusted for potential confounding variables such as age, gender, race, education level, smoking status, alcohol consumption, alanine aminotransferase, aspartate aminotransferase, diabetes, hypertension, BMI, and waist circumference, in line with extensive literature evidence and clinical experience.

The study systematically evaluated the predictive efficacy of plasma TG, HDL-c, and their ratio for MASLD using the Receiver Operating Characteristic (ROC) curve and its Area Under the Curve (AUC). The ROC-AUC values for each lipid index were calculated and compared to determine their relative efficacy in predicting MASLD risk.

To assess the robustness of the results, the following sensitivity analyses were conducted: 1) We addressed missing covariate data through multiple imputation using the multivariate imputation by chained equations (MICE) method, generating 20 imputed datasets. The main analysis was then repeated across all imputed datasets, and results were pooled according to Rubin’s rules to account for both within- and between-imputation variance; 2) stratified analysis in the main population sample to test the stability of the primary findings within each stratum.

All statistical computations were performed using the R programming language (version 4.2.2), with a two-sided *P*-value of <0.05 as the threshold for statistical significance. The original data cleaning and analysis were primarily conducted between October 1 and December 1, 2023.

## 3 Result

**Figure 2:**
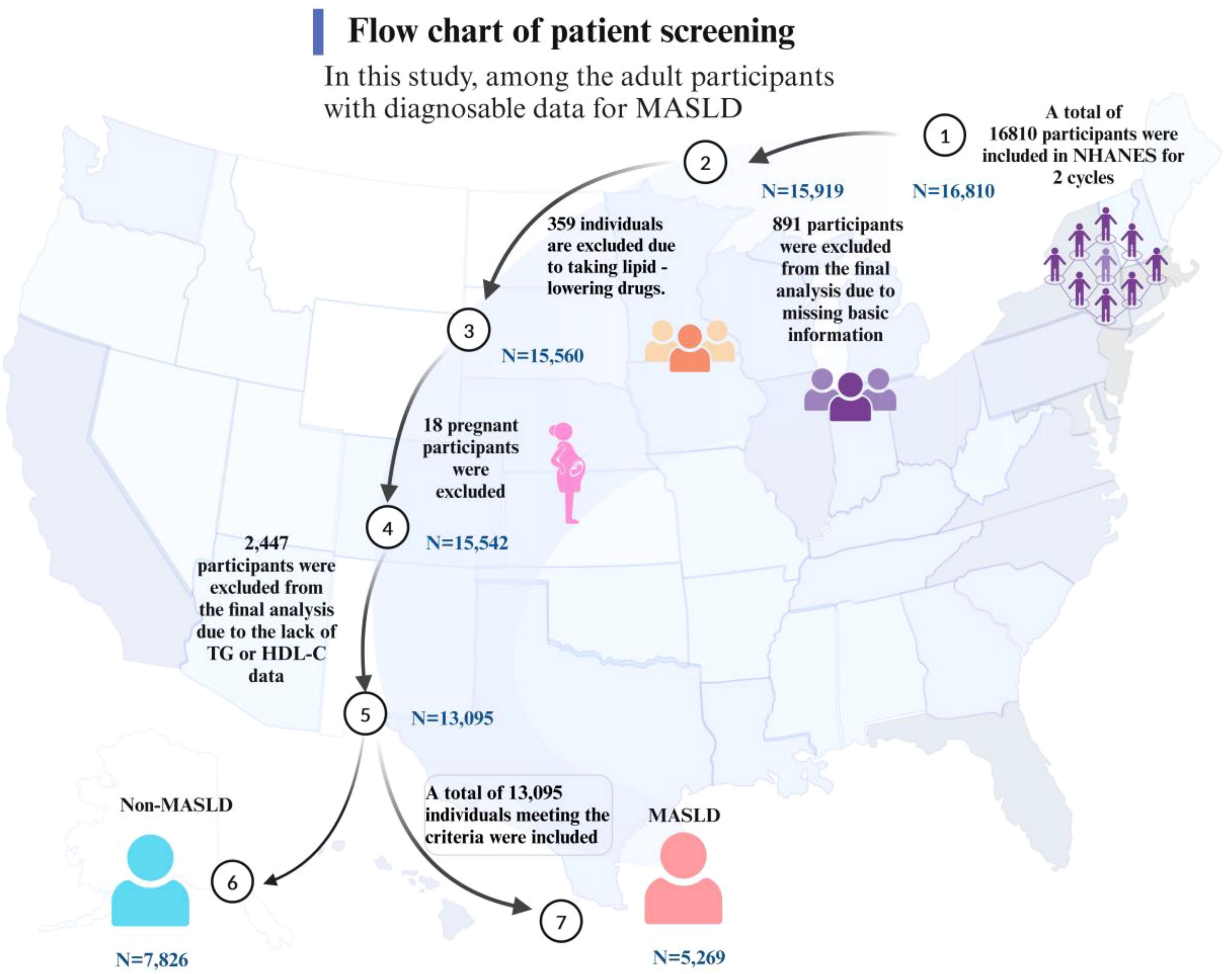
Flow chart of participants.

In this study, among the adult participants with diagnosable data for MASLD, a total of 16,810 individuals meeting the criteria were included, representing an estimated 243 million American adults. Of these, 3,715 participants were excluded from the final analysis due to missing relevant data or failing to meet the inclusion criteria (**Figure 2**). Comparisons revealed that the participants excluded and those included in the final analysis were similar in characteristics such as gender and educational level. However, there were some differences in racial composition, particularly among the African American population, as detailed in **Supplementary Table S1**.

In the overall sample, the mean age was 47.98 years, with a BMI of 29.81 kg/m^2^, and a waist circumference (WC) of 100.72 cm. Females constituted 51.72% of the sample. The racial composition was predominantly non-Hispanic white (62.95%). A history of diabetes was present in 15.74% of the participants. Over half had a higher educational level (60.83%), and 36.05% had a history of hypertension. A majority, 57.77%, never smoked, and only 6.69% abstained completely from alcohol. Compared to the non-MASLD group, participants in the MASLD group were older (50.83 vs 46.09 years), and had higher BMI, WC, and lipid profiles, including TG, TG/HDL-c ratio, low-density lipoprotein cholesterol (LDL-c), alanine aminotransferase (ALT), and aspartate aminotransferase (AST) levels. The MASLD group also had a higher proportion of males (55.96% vs 43.20%), higher prevalence of diabetes (26.46% vs 8.66%), and hypertension (49.42% vs 27.23%). Despite its role as a liver dysfunction marker, total bilirubin levels showed no significant difference between groups, unlike other hepatic enzymes measured (**Table 1**).

**Table 1:** Basic information of participants (Weight).

Variables	Total (n = 13,095)	non-MASLD (n = 7,826)	MASLD (n = 5,269)	P - value
Age, mean (SE), years	47.98 (0.40)	46.09 (0.44)	50.83 (0.42)	< 0.0001
BMI, mean (SE), kg/m^2	29.81 (0.16)	27.02 (0.13)	33.98 (0.21)	< 0.0001
WC, mean (SE), cm	100.72 (0.43)	93.32 (0.40)	111.70 (0.51)	< 0.0001
TG, median (Q1, Q3), mmol/L	1.30 (0.90,1.92)	1.10 (0.80,1.57)	1.68 (1.20,2.35)	< 0.0001
HDL-c, median (Q1, Q3), mmol/L	1.32 (1.09,1.60)	1.42 (1.22,1.71)	1.19 (1.01,1.40)	< 0.0001
TG/HDL-c ratio	0.97 (0.60,1.63)	0.77 (0.49,1.21)	1.40 (0.91,2.19)	< 0.0001
LDL-c, median (Q1, Q3), mmol/L	2.77 (2.22,3.36)	2.69 (2.20,3.28)	2.92 (2.28,3.49)	0.01
ALT, median (Q1, Q3), U/L	18.00 (13.00,26.00)	16.00 (12.00,22.00)	22.00 (16.00,32.00)	< 0.0001
AST, median (Q1, Q3), U/L	19.00 (16.00,24.00)	19.00 (16.00,23.00)	20.00 (16.00,26.00)	< 0.0001
TBIL, median (Q1, Q3), umol/L	6.84 ( 5.13,10.26)	6.84 (5.13,10.26)	6.84 (5.13,10.26)	0.17
Sex (%)				< 0.0001
Female	6,771 (51.72)	4,392 (56.80)	2,379 (44.04)	
Male	6,324 (48.28)	3,434 (43.20)	2,890 (55.96)	
Eth (%)				< 0.0001
Non-Hispanic White	4,610 (62.95)	2,716 (63.16)	1,894 (62.63)	
Non-Hispanic Black	3,176 (10.75)	2,115 (12.10)	1,061 ( 8.71)	
Mexican American	1,671 ( 8.88)	750 ( 6.77)	921 (12.07)	
Other Hispanic	1,335 ( 7.37)	778 (7.65)	557 (6.95)	
Other Race	2,303 (10.05)	1,467 (10.32)	836 ( 9.65)	
DM (%)				< 0.0001
no	10,352 (84.26)	6,782 (91.34)	3,570 (73.54)	
yes	2,743 (15.74)	1,044 (8.66)	1,699 (26.46)	
Education (%)				0.06
low	1,025 (3.68)	556 (3.61)	469 (3.87)	
middle	4,598 (34.67)	2,736 (34.05)	1,862 (36.32)	
high	7,261 (60.83)	4,373 (62.34)	2,888 (59.82)	
Hypertension (%)				< 0.0001
no	7,728 (63.91)	5,156 (72.77)	2,572 (50.58)	
yes	5,355 (36.05)	2,664 (27.23)	2,691 (49.42)	
Smoke (%)				< 0.001
never	7,690 (57.77)	4,689 (58.69)	3,001 (56.41)	
former	3,087 (25.32)	1,643 (23.30)	1,444 (28.39)	
now	2,314 (16.89)	1,491 (18.01)	823 (15.20)	
Alcohol (%)				0.02
never	1,185 (6.69)	728 (8.16)	457 (8.76)	
mild	4,349 (35.76)	2,527 (43.46)	1,822 (47.11)	
moderate	2,029 (17.71)	1,295 (24.24)	734 (19.21)	
heavy	2,214 (19.47)	1,285 (24.15)	929 (24.93)	

BMI, Body Mass Index; WC, waist circumference; TG, triglycerides; HDL-c, high-density lipoprotein cholesterol; LDL-c, low-density lipoprotein cholesterol; ALT, alanine aminotransferase;AST, aspartate aminotransferase; TBIL, total bilirubin; DM, diabetes mellitus.

All estimates accounted for complex survey designs, and all percentages were weighted.

This study assessed the association between plasma TG, HDL-c, and the TG/HDL-c ratio with MASLD. As indicated in **Table 2**, in the univariate model analysis, elevated plasma TG levels (Odds Ratio [OR] = 2.16, 95% Confidence Interval [CI]: 2.02-2.32), reduced HDL-c levels (OR = 0.16, 95% CI: 0.13-0.20), and a higher TG/HDL-c ratio (OR = 1.99, 95% CI: 1.83-2.15) were significantly associated with an increased prevalence rate of MASLD. These associations persisted in the multivariate model adjusted for demographic and metabolic relative factors (TG OR = 1.80; HDL-c OR = 0.60; TG/HDL-c ratio OR = 1.69). Furthermore, these relationships remained stable even after multiple imputation of missing covariates. Therefore, these findings suggest that dyslipidemia indices serve as robust and independent biochemical predictors of MASLD, unaffected by significant confounding from other common relative factors.

**Table 2:** The association between TG, HDL-c, and TG/HDL-c ratio with MASLD (Weight).

	Original data set				Interpolation data set			
Variables	Univariate model		Multivariate model		Univariate model		Multivariate model	
	OR (95%CI)	*P* - value	OR (95%CI)	*P* - value	OR (95%CI)	*P* - value	OR (95%CI)	*P* - value
TG	2.16 (2.02,2.32)	<0.0001	1.80 (1.52,2.12)	<0.0001	2.16 (2.02,2.32)	<0.0001	1.59 (1.47,1.71)	<0.0001
HDL	0.16 (0.13,0.20)	<0.0001	0.60 (0.41,0.86)	<0.0001	0.16 (0.13,0.20)	<0.0001	0.40 (0.30,0.53)	<0.0001
TG/HDL	1.99 (1.83,2.15)	<0.0001	1.69 (1.43,1.99)	<0.0001	1.99 (1.83,2.15)	<0.0001	1.50 (1.39,1.63)	<0.0001

TG, triglycerides; HDL-c, high-density lipoprotein cholesterol.

All estimates accounted for complex survey designs, and all percentages were weighted.

Adjusted Model: age, sex, race, education,smoke, alcohol, ALT, AST, DM, Hypertension, BMI, WC.

**Figure 3:**
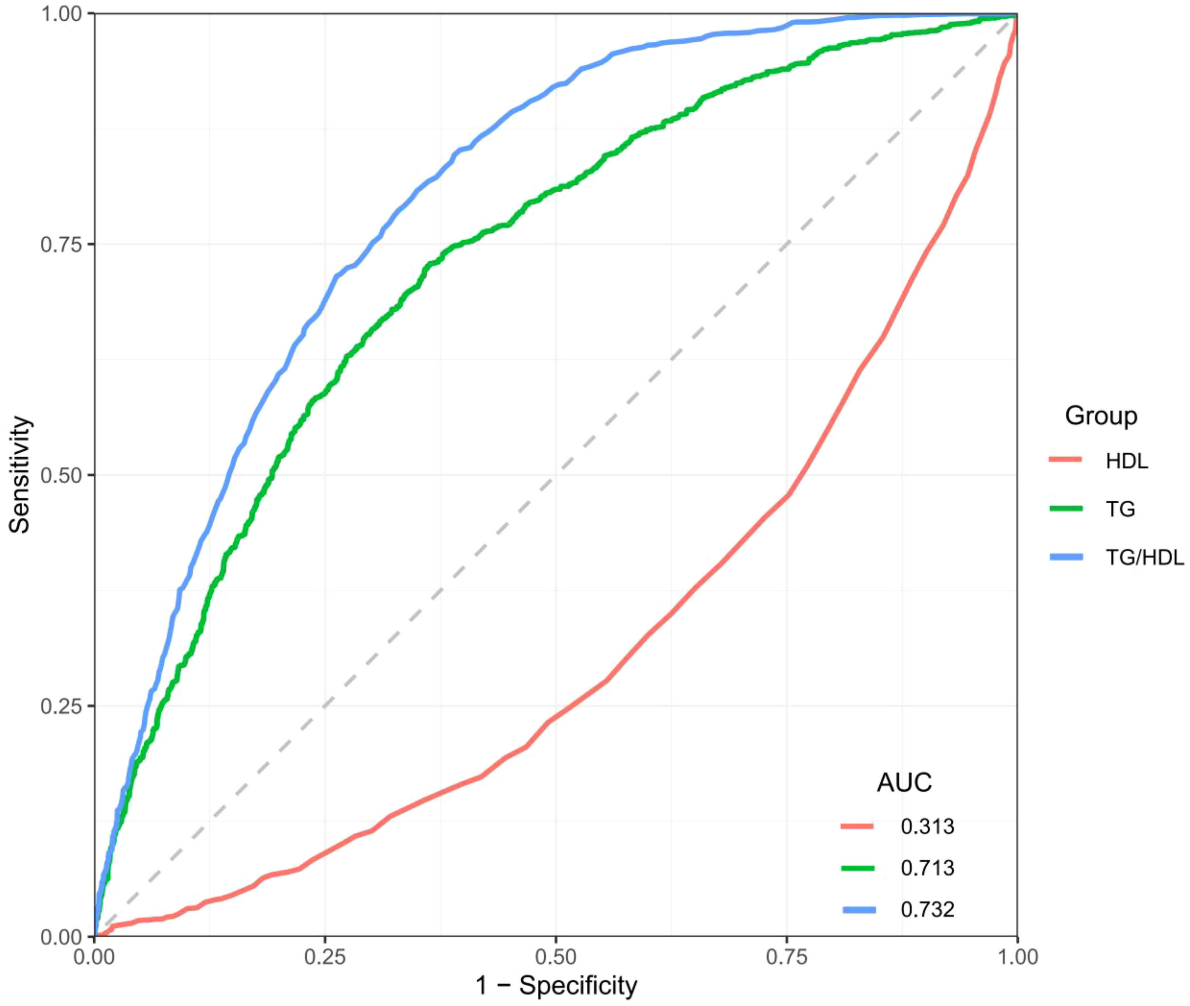
ROC curve comparison of HDL, TG, and TG/HDL ratio for MASLD prediction.

To thoroughly evaluate the effectiveness of plasma TG, high-density HDL-c, and their ratio in predicting the risk of diagnose MASLD, this study utilized ROC curves and calculated the AUC for each. As illustrated in **Figure 3**, the AUC values for predicting MASLD were 0.713 for TG, 0.313 for HDL-c, and 0.732 for the TG/HDL-c ratio (**Supplementary Table S2**). These findings suggest that the TG/HDL-c ratio, which reflects dynamic changes in lipid metabolism, could serve as a practical and accessible screening parameter for MASLD risk assessment in clinical settings, with consideration for demographic factors.

Current research further compared the predictive performance of the TG/HDL-c ratio with traditional clinical indicators including BMI, FPG, and liver enzymes. The ROC analysis showed varying predictive capabilities among these markers (AUC values: BMI = 0.647, FPG = 0.609, GGT = 0.493, ALT = 0.370, AST = 0.338), with TG/HDL-c ratio demonstrating superior performance (AUC = 0.732) for MASLD. ROC analysis identified optimal cutoff values: TG/HDL-c ratio at 0.80 achieved optimal diagnostic performance (sensitivity 75%, specificity 71%, Youden index 0.46), outperforming other indicators including BMI (25 kg/m², sensitivity 75%, specificity 70%), FPG (5.6 mmol/L, sensitivity 70%, specificity 72%), GGT (50 U/L, sensitivity 62%, specificity 60%), ALT (30 U/L, sensitivity 65%, specificity 55%), and AST (20 U/L, sensitivity 60%, specificity 58%) (**Supplementary Table S3**, **Supplementary Figure S1**).

To ensure robustness in the results, this study incorporated a multi-dimensional demographic index and employed a stratified sensitivity analysis strategy. Stratification by age, gender, race, and physical activity was conducted to account for these factors, which are known to influence both lipid metabolism and the risk of MASLD. These stratifications allowed us to assess whether the predictive value of TG/HDL-c ratio differed across subgroups, providing a more comprehensive understanding of the biomarkers’ relevance in different populations. Additional sensitivity analyses adjusting for physical activity levels confirmed that the association between TG/HDL-c ratio and MASLD remained significant (OR = 1.67, 95% CI: 1.41-1.97, *P* < 0.001), indicating the stability of this relationship independent of exercise habits. Moreover, the use of the AUC as a predictive method provided a quantitative and intuitive display of the consistency in the efficacy of the three lipid indices in predicting MASLD across all stratified population groups. The study systematically found that, regardless of the population stratification configuration, the association and predictive efficacy of plasma lipid abnormalities with MASLD were consistently replicable with the results of the original total population analysis (**Supplementary Table S4**, **Supplementary Figures S2**-**S5** for details). This validation of predictive indicator stability supports its broad applicability in the risk assessment and screening of metabolic associated fatty liver disease in various populations.

## 4 Discussions

### 4.1 Main findings

This study evaluated the association between plasma TG, HDL-c, and their ratio with MASLD in a sample of the normal adult population in the U.S. The results indicated that, both in univariate and multivariate model analyses, increased levels of plasma TG, decreased levels of HDL-c, and a higher TG/HDL-c ratio were significantly associated with MASLD. This suggests that dyslipidemia can serve as an independent and robust predictive marker for MASLD, with its predictive value not significantly affected by other common related factors.

Furthermore, to systematically evaluate the predictive efficacy of plasma TG, HDL-c, and their ratio for MASLD, the study employed ROC curves and the AUC method. The results demonstrated that the TG/HDL-c ratio, reflecting dynamic changes in lipid metabolism, had a higher predictive efficacy than individual lipid indices. This confirmed the clinical application value of TG/HDL-c ratio as an independent and robust predictive marker for MASLD.

For assessing the robustness of the results, the study utilized a stratified analysis approach, dividing the U.S. adult population into subgroups based on demographic characteristics and examining the consistency of the main findings within each group. It was found that across all U.S. adult population stratifications, the association and predictive efficacy of TG, HDL-c, and their ratio with MASLD were stable, with the predictive efficacy of TG/HDL-c ratio superior to that of TG or HDL-c in all subgroups. This supports the wide applicability of this predictive marker in the risk assessment and screening for MASLD across various U.S. adult populations.

The clinical significance of these findings lies in the potential of the TG/HDL-c ratio as an easily accessible and cost-effective biomarker for MASLD. Given its superior predictive efficacy compared to TG or HDL-c alone, the TG/HDL-c ratio could be incorporated into routine clinical practice and population screening programs to identify individuals at higher risk for MASLD. This could facilitate early detection and timely interventions, particularly in populations at high risk for metabolic dysfunction and fatty liver disease. Moreover, using the TG/HDL-c ratio as a screening tool could help streamline resource allocation for more invasive diagnostic procedures, such as liver biopsy, by identifying those who are more likely to benefit from further investigation and management.

### 4.2 Comparison with literature

Existing literature has explored TG/HDL-c ratio’s predictive value for fatty liver disease. Fan et al. found that TG/HDL-c ratio was significantly associated with NAFLD, reporting gender-specific cutoff values(0.9 for women, 1.4 for men) with AUC values of 0.85 in women and 0.79 in men. In contrast, this study identified a unified cutoff of 0.8 for MASLD prediction, with comparable diagnostic performance (AUC = 0.732). Similarly, Ma et al. conducted a large-scale retrospective cohort study and confirmed that the TG/HDL-c ratio is an independent predictive factor for MAFLD, with higher ratios associated with increased risk. This difference in cutoff values may reflect the distinct pathophysiological mechanisms between NAFLD and MASLD, particularly given MASLD’s stronger association with metabolic dysfunction. Additionally, while Fan et al. observed notable gender differences in predictive performance, this study analysis found more consistent predictive value across gender groups. These variations might be attributed to differences in study populations and the evolving understanding of fatty liver disease classification from NAFLD to MASLD (32). Another study in a non-obese Chinese population with normal blood lipid levels showed similar findings, suggesting a gender difference in the assessment of MASLD using TG/HDL-c ratio (male AUC 0.69 vs. female AUC 0.65) (33). A 10-year longitudinal cohort study in Japan also indicated a gender difference in predicting MASLD with this ratio (male AUC 0.88 vs. female AUC 0.64) (34). This differs slightly from this study, where this study did not observe a significant difference in predictive efficacy between genders (male AUC 0.726 vs. female AUC 0.718). The smaller sample sizes of these cohort studies, compared to the present larger sample (n=14,148, representative of 243 million US adults when weighted), might be a reason for these differences. The diverse racial composition and lifestyle differences across study populations suggest that both genetic and environmental factors may significantly influence our findings. Additionally, another study using TG/HDL-c ratio to predict MASLD reported an AUC of 0.645 (35), further indicating the need for independent research in different populations to explore the relationship. Overall, both the current study and those of other researchers confirm a close association between TG/HDL-c ratio and MASLD. As TG/HDL-c ratio levels increase, the risk of developing MASLD rises.

Moreover, numerous studies have validated the efficacy of the TG/HDL-c ratio in predicting various other diseases. For instance, an analysis by Liu et al. (34) of inpatient data from a university hospital in China found a significant positive association between TG/HDL-c ratio levels and insulin resistance, suggesting its potential as an alternative biochemical marker for assessing insulin resistance. In populations with obesity-related hypertension, studies have confirmed that higher TG/HDL-c ratio is independently associated with an increased risk of hypertension (36). Additionally, Chen and colleagues, analyzing data from a large prospective cohort of Chinese adults, reported that a higher TG/HDL-c ratio independently predicts an increased risk of cardiovascular events (37). Statistical analyses of the UK Biobank data also indicate that TG/HDL-c ratio may be a potential risk factor for cardiovascular disease. A recent study by Cai et al. (33) suggested that increased variability in TG/HDL-c ratio could predict a higher incidence of diabetes in the Chinese adult population, regardless of the direction of variability. Furthermore, other studies have shown that TG/HDL-c ratio is also a potentially effective biochemical marker for predicting risks of metabolic syndrome, hyperuricemia, and chronic kidney disease.

### 4.3 Mechanisms

Although the precise mechanisms linking TG/HDL-c ratio with MASLD are yet to be fully elucidated, existing research offers several potential explanations. Insulin resistance (IR) is considered a key factor in the development and progression of MASLD (38, 39). An increase in the TG/HDL-c ratio has been established as a surrogate marker of IR, playing a central role in the pathogenesis of MASLD (40). For example, hypertrophied adipose tissue secretes cytokines that affect IR, including leptin, adiponectin, and various inflammatory factors, which can promote the development of hepatic IR (41–43). IR, in turn, affects lipoprotein metabolism by promoting the secretion of triglyceride-enriched very-low-density lipoproteins (VLDL), and inhibits the expression of anti-inflammatory adiponectin, leading to reduced serum HDL-c (44, 45). This results in an increased serum TG/HDL-c ratio. Additionally, other cell types such as Kupffer cells and stellate cells might influence hepatocyte status by promoting hepatic lipid accumulation or inhibiting triglyceride breakdown (46, 47). Overall, the TG/HDL-c ratio may reflect a series of imbalances in hepatic lipid synthesis, transport, and degradation processes, as well as cytokine-mediated lipid accumulation and inflammatory damage. This could be a key mechanism underlying its strong association with MASLD.

## 5 Strengths and limitations

Those findings has several strengths. One of the key strengths is the large sample size, which enhances the generalizability of the findings to the broader population. Additionally, the comprehensive nature of the data collection allows for a robust assessment of the relationship between TG/HDL-c ratio and MASLD.

However, there are certain limitations to acknowledge. Firstly, the coverage of This research population is limited, as specific groups such as pregnant women and children were not included. Whether the current findings are applicable to these populations remains to be verified. Nevertheless, this study are continuously collecting relevant data and anticipate addressing this limitation in the near future. Secondly, as a cross-sectional study, this study cannot establish a causal relationship between TG/HDL-c ratio and MASLD and can only assess its utility in aiding diagnosis. To address this, we are planning a prospective cohort study to clarify the cause-and-effect relationship in the future. Furthermore, for certain diseases like diabetes, we have not yet specified subtypes in detail. However, given the large sample size of the data analysis, this is unlikely to have a significant impact on the main outcomes.

## 6 Conclusion

This study utilized the NHANES database to demonstrate that elevated TG, reduced HDL-c, and particularly a higher TG/HDL-c ratio are independently associated with MASLD in a representative U.S. population. The TG/HDL-c ratio showed stronger predictive value than individual lipid markers, suggesting its utility as a simple, cost-effective screening tool in clinical practice. This readily available marker could enable early risk stratification and targeted interventions during routine patient care. Future studies should focus on validating optimal TG/HDL-c ratio cutoff points for different populations and evaluating whether ratio-guided screening strategies improve patient outcomes.

## Data Availability

Data described in the manuscript are publicly available at the NHANES website: https://www.cdc.gov/nchs/nhanes/index.html; The analytic code described in the manuscript will be made available upon request pending application and approval from the corresponding author.
