# Supplementary material for: Triglycerides/high-density lipoprotein-cholesterol ratio outperforms traditional lipid indicators in predicting metabolic dysfunction-associated steatotic liver disease among U.S. adults
Source: Front Endocrinol (Lausanne). 2025 Apr 15;16:1591241. doi: 10.3389/fendo.2025.1591241 (PMC12037621; doi:10.3389/fendo.2025.1591241)
Supplement: Supplementary file 1 [file DataSheet1.docx]

**Supplementary material**

**Attachment 1. Similarity Report**

**Attachment 2. Certificate of language editing**

**Supplementary table legends**

**Table S1. Comparison between inclusion and missing**

**Table S2. Comparison of subgroup ROC curves**

**Table S3. Diagnostic Performance of Various Biomarkers for MASLD Prediction**

**Table S4. Sensitivity analysis of associations between lipid indicators and MASLD after adjusting for physical activity**

**Table S5. The association between different subgroups of TG, HDL-c and TG/HDL-c ratio and MASLD(Weight)**

| **Table S1. Comparison between inclusion and missing** | | | | |
| --- | --- | --- | --- | --- |
| **Variables** | **Missing values** | | **Select values** | |
|  | **n = 3,715** | **per** | **n = 13,095** | **per** |
| **Eth** |  |  |  |  |
| Mexican American | 223 | 0.06 | 1,671 | 0.13 |
| Black | 1,486 | 0.4 | 3,176 | 0.24 |
| White | 892 | 0.24 | 4,610 | 0.35 |
| Hispanic | 334 | 0.09 | 1,335 | 0.1 |
| Other | 780 | 0.21 | 2,303 | 0.18 |
| **Sex** |  |  |  |  |
| Male | 1,820 | 0.49 | 6,324 | 0.48 |
| Female | 1,895 | 0.51 | 6,771 | 0.52 |
| **Edu** |  |  |  |  |
| high | 1,969 | 0.53 | 7,261 | 0.55 |
| middle | 1,337 | 0.36 | 4,598 | 0.35 |
| low | 223 | 0.06 | 1,025 | 0.08 |

| **Table S2. Comparison of subgroup ROC curves** | | | | |
| --- | --- | --- | --- | --- |
| **Varibles** | | **AUC** | | |
|  |  | **TG** | **HDL** | **TG/HDL** |
| **Age** | |  |  |  |
| <40 | | 0.756 | 0.214 | 0.787 |
| 40~60 | | 0.68 | 0.313 | 0.703 |
| >60 | | 0.675 | 0.354 | 0.689 |
| **Sex** | |  |  |  |
| Male | | 0.715 | 0.322 | 0.726 |
| Female | | 0.701 | 0.327 | 0.718 |
| **Eth** | |  |  |  |
| Non-Hispanic Black | | 0.675 | 0.358 | 0.689 |
| Non-Hispanic White | | 0.724 | 0.309 | 0.742 |
| Mexican American | | 0.689 | 0.279 | 0.721 |
| Other Hispanic | | 0.701 | 0.336 | 0.712 |
| Other Race | | 0.691 | 0.331 | 0.708 |
| **Education** | |  |  |  |
| low | | 0.671 | 0.358 | 0.68 |
| middle | | 0.691 | 0.341 | 0.713 |
| high | | 0.723 | 0.293 | 0.745 |
| note: All estimates accounted for complex survey designs, and all percentages were weighted. | | | | |

| **Table S3. Diagnostic Performance of Various Biomarkers for MASLD Prediction** | | | | | | |  |
| --- | --- | --- | --- | --- | --- | --- | --- |
| **Biomarker** | **AUC** | **95% CI** | **Cut-off Value** | **Sensitivity** | **Specificity** | **Youden Index** | |
| TG/HDL-c | 0.732 | [0.683, 0.781] | 0.8 | 0.75 | 0.71 | 0.46 | |
| TG | 0.713 | [0.664, 0.762] | 1.71 | 0.71 | 0.7 | 0.41 | |
| HDL-c | 0.313 | [0.264, 0.362] | 1.82 | 0.6 | 0.58 | 0.18 | |
| BMI | 0.647 | [0.597, 0.697] | 25 | 0.75 | 0.7 | 0.45 | |
| FPG | 0.61 | [0.560, 0.660] | 5.6 | 0.7 | 0.72 | 0.42 | |
| GGT | 0.493 | [0.443, 0.543] | 50 | 0.62 | 0.6 | 0.22 | |
| ALT | 0.37 | [0.320, 0.420] | 30 | 0.65 | 0.55 | 0.2 | |
| AST | 0.338 | [0.288, 0.388] | 20 | 0.6 | 0.58 | 0.18 | |
| Note: Biomarkers are arranged by category (lipid markers, anthropometric measure, glycemic marker, and liver enzymes) and ordered by AUC within each category. AUC: Area Under the Curve; CI: Confidence Interval; TG: Triglycerides; HDL-c: High-density Lipoprotein Cholesterol; BMI: Body Mass Index; FPG: Fasting Plasma Glucose; GGT: Gamma-glutamyl Transferase; ALT: Alanine Aminotransferase; AST: Aspartate Aminotransferase. | | | | | | | |

| **Table S4. Sensitivity analysis of associations between lipid indicators and MASLD after adjusting for physical activity** | | | | |
| --- | --- | --- | --- | --- |
| **Varibles** | **Original data set** | | **Interpolation data set** | |
|  | **Multivariate model** | | **Multivariate model** | |
|  | **OR(95%CI)** | ***P*** - value | **OR(95%CI)** | ***P*** - value |
| TG | 1.79(1.49,2.09) | <0.0001 | 1.58(1.46,1.70) | <0.0001 |
| HDL | 0.63(0.42,0.88) | <0.0001 | 0.42(0.32,0.52) | <0.0001 |
| TG/HDL | 1.67(1.41,1.7) | <0.0001 | 1.53(1.42,1.66) | <0.0001 |
| Abbreviate:TG, triglycerides; HDL-c, high-density lipoprotein cholesterol | | | | |
| note: All estimates accounted for complex survey designs, and all percentages were weighted. | | | | |
| Adjusted Model: age, sex, race, education,smoke, alcohol, ALT, AST, DM, Hypertension, BMI, WC, exercise | | | | |

| **Table S5. The association between different subgroups of TG, HDL-c and TG/HDL-c ratio and MASLD(Weight)** | | | | | | |
| --- | --- | --- | --- | --- | --- | --- |
| **Varibles** | **TG** | | **HDL-c** | | **TG/HDL-c** | |
|  | **OR(95%CI)** | P - value | **OR(95%CI)** | P - value | **OR(95%CI)** | P - value |
| **Age** |  |  |  |  |  |  |
| <40 | 1.770(1.553,2.018) | <0.0001 | 0.099(0.055,0.177) | <0.0001 | 1.761(1.543,2.009) | <0.0001 |
| 40~60 | 1.723(1.450,2.047) | <0.0001 | 0.362(0.238,0.550) | <0.0001 | 1.603(1.351,1.902) | <0.0001 |
| >60 | 1.733(1.474,2.038) | <0.0001 | 0.573(0.354,0.928) | 0.025 | 1.553(1.322,1.825) | <0.0001 |
| **Sex** |  |  |  |  |  |  |
| Female | 1.839(1.671,2.024) | <0.0001 | 0.450(0.313,0.647) | <0.0001 | 1.795(1.599,2.015) | <0.0001 |
| Male | 1.665(1.495,1.855) | <0.0001 | 0.343(0.220,0.534) | <0.0001 | 1.512(1.370,1.669) | <0.0001 |
| **Eth** |  |  |  |  |  |  |
| Non-Hispanic Black | 1.877(1.587,2.221) | <0.0001 | 0.666(0.483,0.919) | 0.015 | 1.785(1.478,2.157) | <0.0001 |
| Non-Hispanic White | 1.713(1.533,1.915) | <0.0001 | 0.493(0.311,0.783) | 0.004 | 1.577(1.394,1.784) | <0.0001 |
| Mexican American | 1.454(1.167,1.811) | 0.002 | 0.128(0.059,0.279) | <0.0001 | 1.443(1.161,1.793) | 0.002 |
| Other Hispanic | 1.674(1.382,2.028) | <0.0001 | 0.228(0.135,0.385) | <0.0001 | 1.605(1.391,1.852) | <0.0001 |
| Other Race | 1.680(1.398,2.019) | <0.0001 | 0.234(0.130,0.421) | <0.0001 | 1.540(1.229,1.931) | <0.001 |
| **Education** |  |  |  |  |  |  |
| low | 1.446(1.178,1.776) | <0.001 | 0.518(0.227,1.185) | 0.115 | 1.325(1.121,1.566) | 0.002 |
| middle | 1.561(1.386,1.758) | <0.0001 | 0.424(0.245,0.734) | 0.003 | 1.443(1.292,1.611) | <0.0001 |
| high | 1.921(1.732,2.131) | <0.0001 | 0.319(0.251,0.404) | <0.0001 | 1.870(1.668,2.095) | <0.0001 |
| Abbreviate:TG, triglycerides; HDL-c, high-density lipoprotein cholesterol | | | | | | |
| note: All estimates accounted for complex survey designs, and all percentages were weighted. | | | | | | |

**Supplementary figure legends**

**Figure S1. ROC curves for ALT, AST, GGT, FPG and BMI as predictive markers**

**Figure S2. ROC Curve about different age group**

**Figure S3. ROC Curve about different sex group**

**Figure S4. ROC Curve about different eth group**

**Figure S5. ROC Curve about different education group**

**Figure S1. ROC curves for ALT, AST, GGT, FPG and BMI as predictive markers**


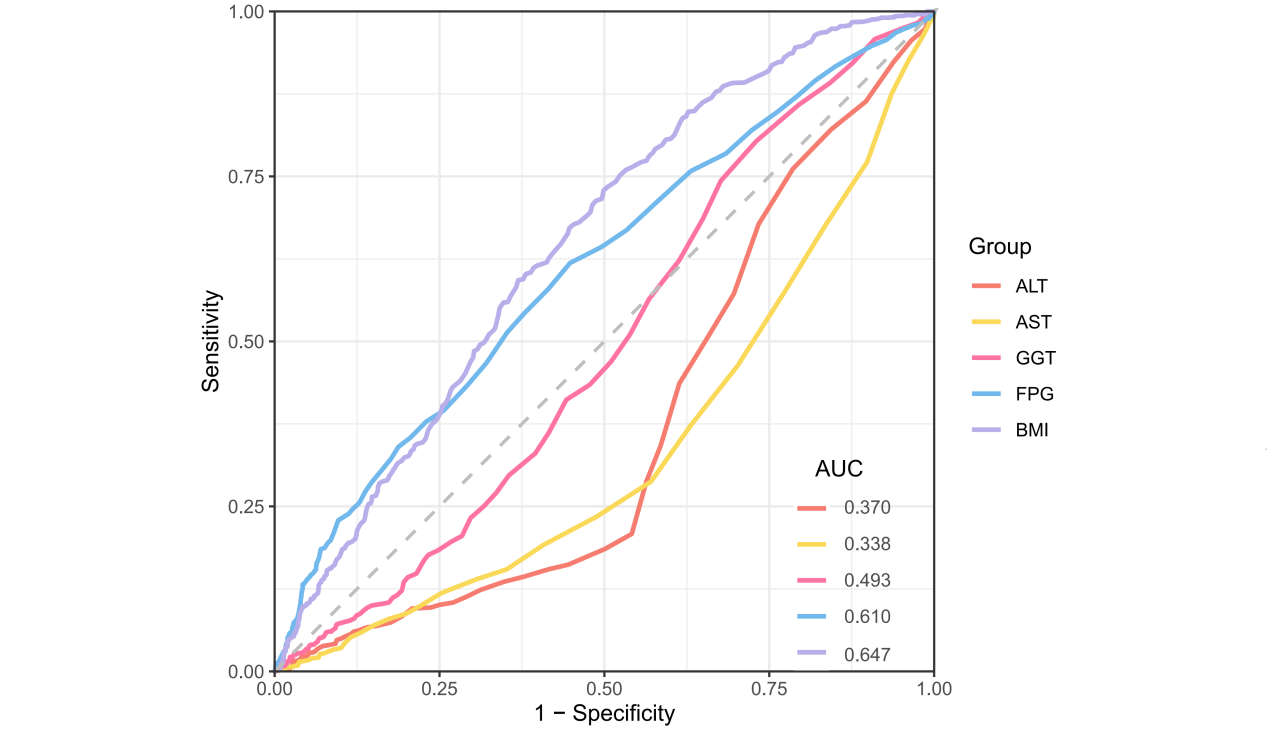


Figure S1. Receiver Operating Characteristic (ROC) Curves for ALT, AST, GGT, FPG and BMI in MASLD Prediction. The graph shows ROC curves comparing different biomarkers, where BMI (AUC = 0.647) and FPG (AUC = 0.61) showed better discriminative ability than GGT (AUC = 0.493), ALT (AUC = 0.37), and AST (AUC = 0.338). The dashed line represents the reference line of no discrimination (AUC = 0.5).

**Figure S2. ROC Curve about different age group**

**
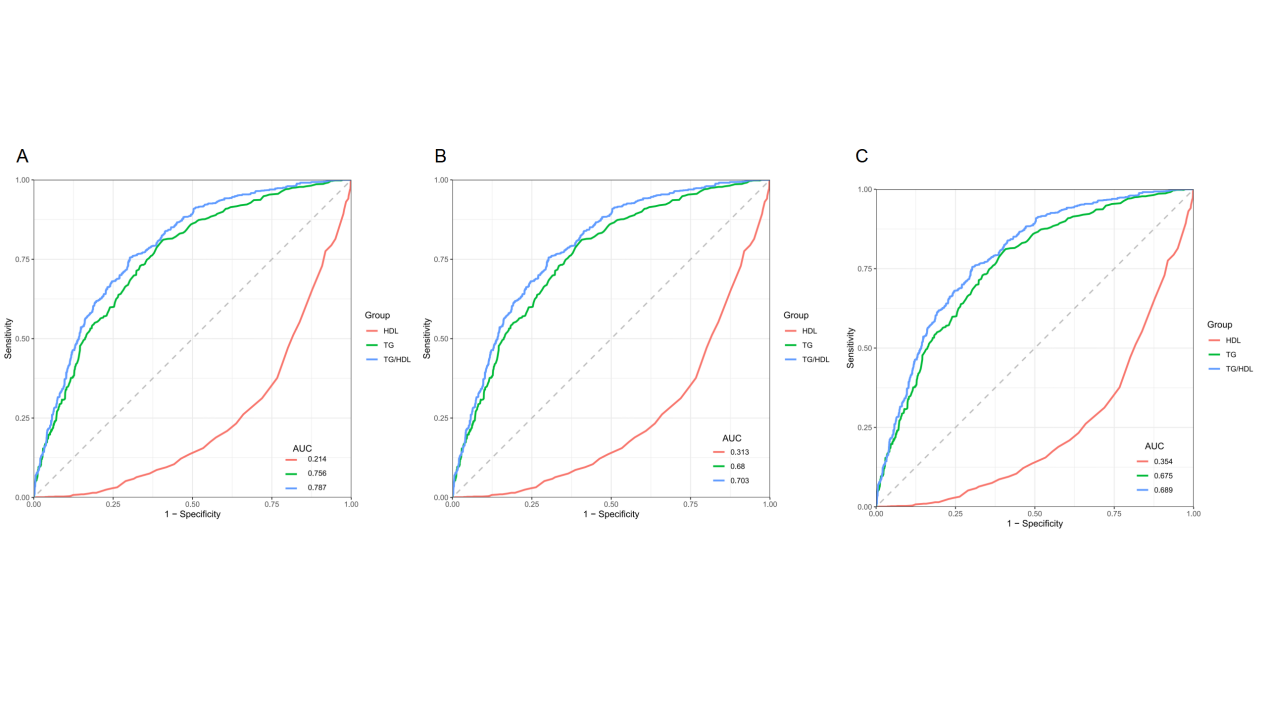
**

Figure S2: Receiver Operating Characteristic (ROC) Curves for Lipid Profile Biomarkers in Predictive Analysis. Panels red, green, and blue display the ROC curves for MASLD assessing the predictive power of HDL, TG, and the TG/HDL-c as biomarkers for cardiovascular risk stratification. The area under the curve (AUC) values are indicated for each biomarker in the respective panels, with values closer to 1.0 suggesting better discriminative ability. The dashed line represents the line of no-discrimination, which corresponds to an AUC of 0.5. A: Age group under 40 years old; B: Age group of 40 to 60 years old; C: Age group over 60 years old.

.

**Figure S3. ROC Curve about different sex group**

**
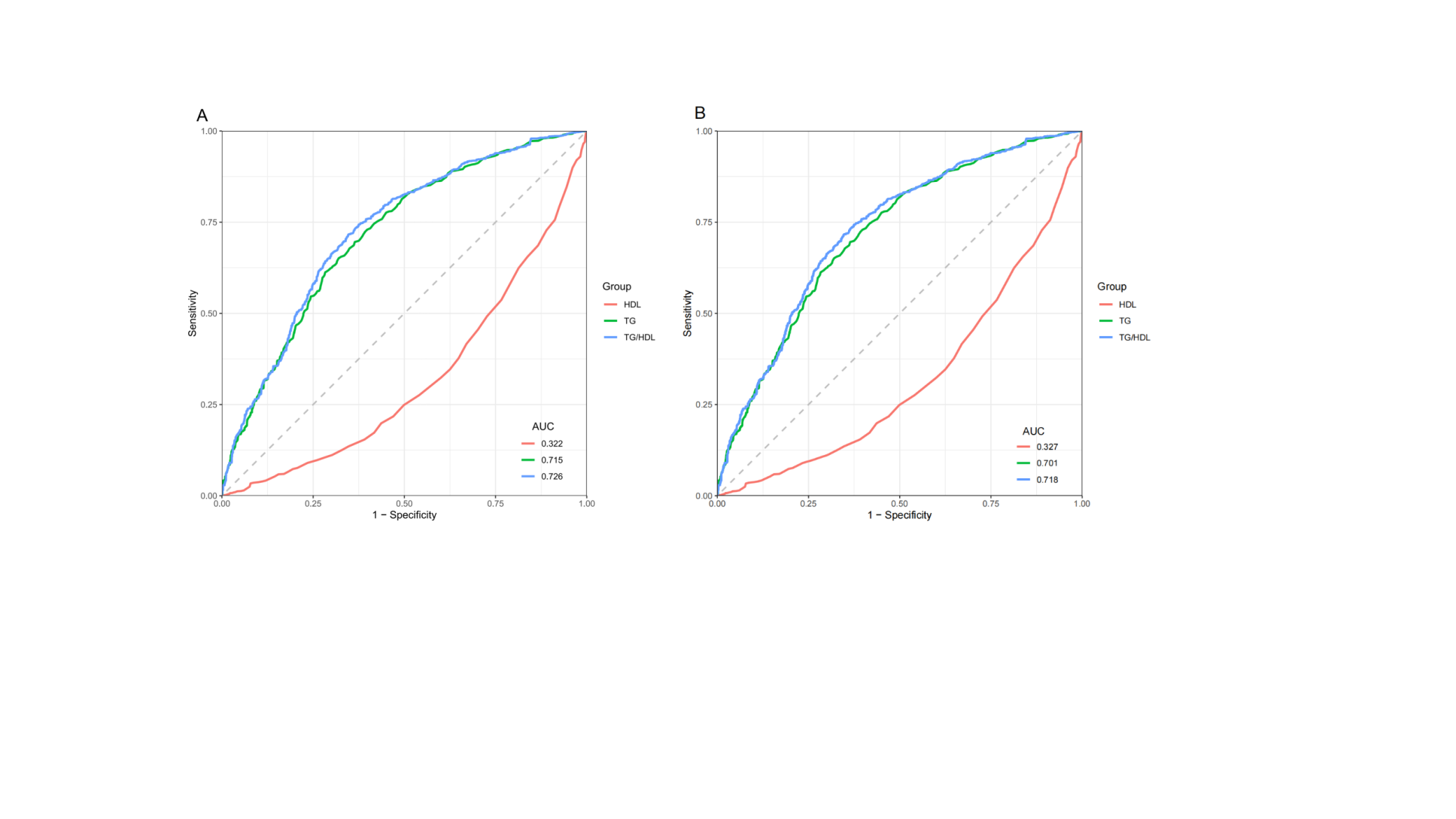
**

Figure S3: Receiver Operating Characteristic (ROC) Curves for Lipid Profile Biomarkers in Predictive Analysis. Panels red, green, and blue display the ROC curves for assessing the predictive power of HDL, TG, and the TG/HDL-c as biomarkers for cardiovascular risk stratification. The area under the curve (AUC) values are indicated for each biomarker in the respective panels, with values closer to 1.0 suggesting better discriminative ability. The dashed line represents the line of no-discrimination, which corresponds to an AUC of 0.5. A: Male group; B: Female group.

.

**Figure S4. ROC Curve about different eth group**


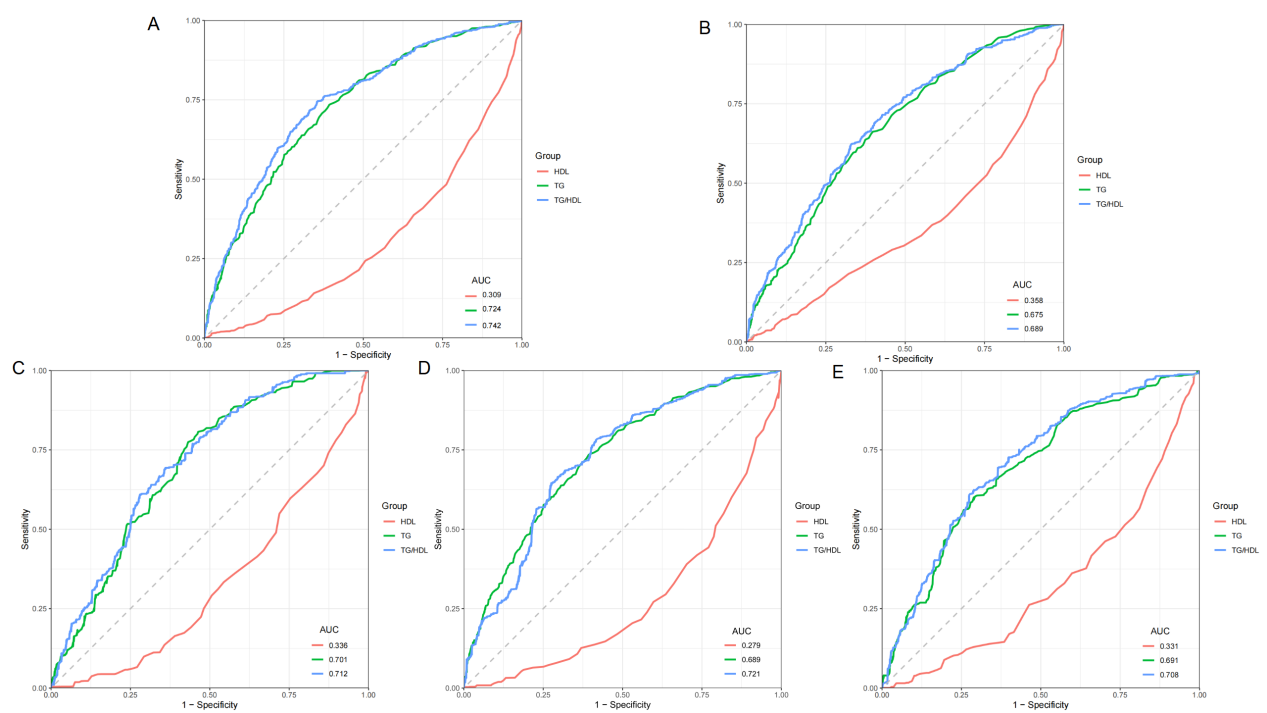


Figure S4: Receiver Operating Characteristic (ROC) Curves for Lipid Profile Biomarkers in Predictive Analysis. Panels red, green, and blue display the ROC curves for MASLD assessing the predictive power of HDL, TG, and the TG/HDL-c as biomarkers for cardiovascular risk stratification. The area under the curve (AUC) values are indicated for each biomarker in the respective panels, with values closer to 1.0 suggesting better discriminative ability. The dashed line represents the line of no-discrimination, which corresponds to an AUC of 0.5. A: Non-Hispanic White; B: Non-Hispanic Black; C: Other Hispanic; D: Mexican American; E: Other Race.

**Figure S5. ROC Curve about different education group**


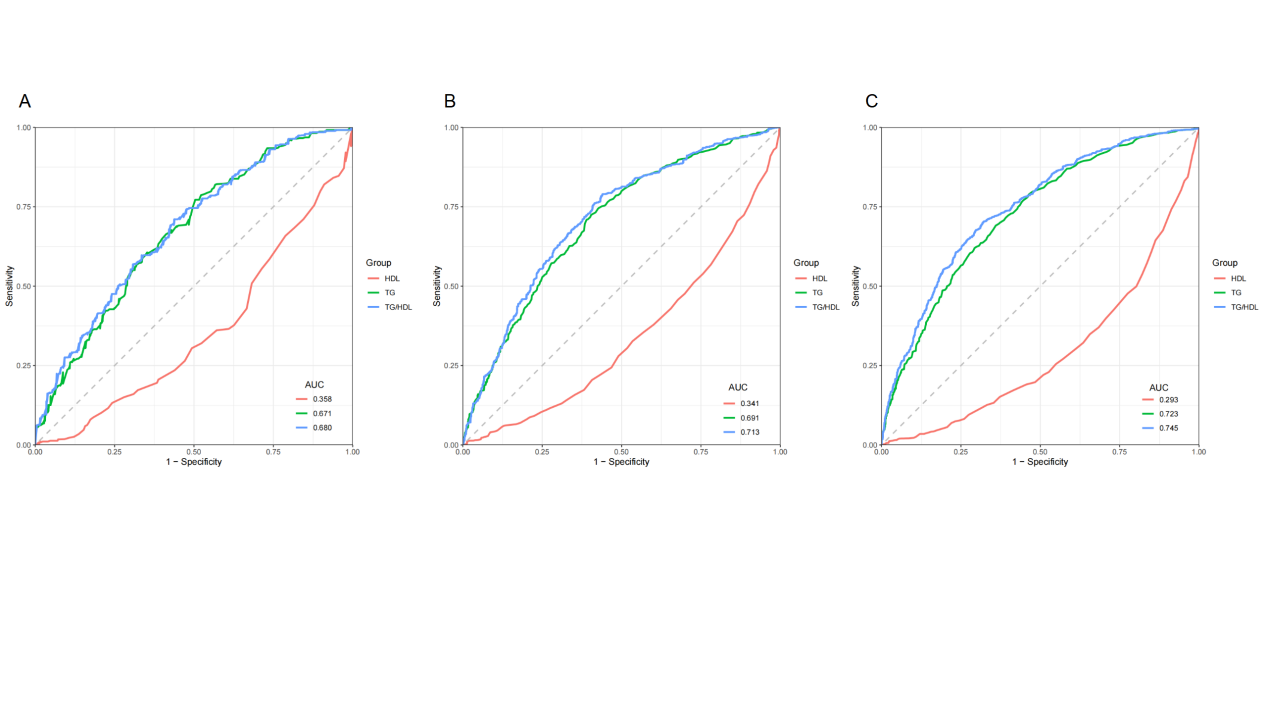


Figure S5: Receiver Operating Characteristic (ROC) Curves for Lipid Profile Biomarkers in Predictive Analysis. Panels red, green, and blue display the ROC curves for MASLD assessing the predictive power of HDL, TG, and the TG/HDL-c as biomarkers for cardiovascular risk stratification. The area under the curve (AUC) values are indicated for each biomarker in the respective panels, with values closer to 1.0 suggesting better discriminative ability. The dashed line represents the line of no-discrimination, which corresponds to an AUC of 0.5. A: Low Education Group; B: Middle Education Group; C: High Education Group.
